# Association of lysyl oxidase-like 1 gene polymorphisms with exfoliation syndrome in Koreans

**Published:** 2011-10-28

**Authors:** Min Sagong, Byoung Young Gu, Soon Cheol Cha

**Affiliations:** Department of Ophthalmology, Yeungnam University College of Medicine, Daegu, Korea

## Abstract

**Purpose:**

To evaluate the association of the lysyl oxidase-like 1 (*LOXL1*) single nucleotide polymorphisms (SNPs) in the Korean population with exfoliation syndrome (XFS) and to investigate the association between the SNPs and phenotypes of XFS.

**Methods:**

Eighty-nine unrelated patients with XFS and 146 unrelated control subjects were recruited. *LOXL1* SNPs, rs1048661, rs3825942, and rs2165241, were genotyped by direct DNA sequencing. Association between cases and controls was analyzed and phenotypic features of XFS were compared in terms of the SNPs.

**Results:**

The three SNPs were found to be significantly associated with XFS. After adjusting for rs3825942, rs2165241, and other factors influencing the prevalence of XFS, only rs1048661 among three SNPs remained significant (95% confidence interval=4.11–35.78, p=6.11×10^−6^). T allele and TT genotype of rs1048661 and C allele and CC genotype of rs2165241 were associated with XFS, showing risk alleles and genotypes opposite to those reported in Caucasians. In the haplotype analysis, T-G-C was the only risk haplotype (p=3.35×10^−12^), which was not associated with XFS in Caucasians. No significant differences were noted in the allele and genotype frequencies depending on phenotypic features of XFS.

**Conclusions:**

Three *LOXL1* SNPs are associated with XFS in the Korean population. Risk alleles and genotypes of rs1048661 and rs2165241 in Korean have a similar pattern with those of East Asians, including Japanese and Northern Chinese, while they have a different pattern from those of Caucasians.

## Introduction

Exfoliation syndrome (XFS) is a complex, late-onset, generalized disorder of the extracellular matrix characterized by production and progressive accumulation of abnormal fibrillar aggregates in various intraocular and extraocular tissues [1,2]. Clinically, XFS is characterized by deposition of white flake-like material on all tissues of the anterior segment of the eye [3]. In particular, gradual accumulations of this material in the outflow pathways may cause a common and severe type of chronic open-angle glaucoma. XFS is acknowledged as the most common identifiable cause of open-angle glaucoma, accounting for about 25% of all open-angle glaucoma worldwide [4]. The prevalence of XFS varies widely among different ethnic populations. In people aged 60 years or older, XFS has been reported to be more common in the Caucasian population with a prevalence of 10%–20% in Northern Europeans [5-8], when compared with the Asian population with a prevalence of 0.4% in Hong Kong Chinese, 0.7% in Singaporean, and 2.4% in Japanese [9-11].

Although the etiology and pathogenesis of XFS are still unknown, recent molecular biologic and biochemical data strongly support XFS as an elastic microfibrillopathy associated with excessive production and abnormal aggregation of elastic fiber components, enzymatic cross-linking processes, and impaired protection mechanisms against oxidative and cellular stress [12-15]. And several population-based and pedigree-based studies have shown that genetic factors may play an important role in the pathogenesis of XFS [16,17].

Recently, a genome-wide association study in the Icelandic and Swedish population demonstrated a significant association of one intronic single nucleotide polymorphisms (SNP, rs2165241) and two exonic SNPs (rs1048661 [R141L] and rs3825942 [G153D]), which are located in the first exon of the lysyl oxidase-like 1 (*LOXL1*) gene on chromosomal region 15q24.1, with XFS and exfoliation glaucoma (XFG) [18]. LOXL1 is a member of the lysyl oxidase family of enzymes, which catalyze covalent cross-linking of collagen and elastin in connective tissues through oxidative deamination [19]. A recent experimental study demonstrated that differential regulation of ocular expression of LOXL1 is dependent on the phase of progression of the fibrotic exfoliation process, contributing to the pathogenesis of XFS [20]. Thus, the hypothesis that defects in *LOXL1* may cause XFS is biologically reasonable.

Association between XFS and the three *LOXL1* SNPs has been confirmed in many Caucasian populations including European, American, and Australian [21-25]. However, in the Asian population, this association has been evaluated only in Indian, Chinese, and Japanese [26-31]. Allelic and genotypic distributions of the three SNPs were found to be drastically different among different ethnic populations. The allelic distribution of East Asian populations including Japanese and Northern Chinese are reversed for rs1048661 and rs2165241 when compared with that of Caucasian populations [26-29]. The G allele of rs1048661 and the T allele of rs2165241, identified as risk alleles in Caucasians, were found to be protective in East Asians. Of particular Interest, genetic diversity also occurs across different districts and populations even in Asia. The Indian population showed risk alleles and genotypes similar to those of Caucasians at rs1048661 and rs2165241 [30]. In the Singaporean Chinese population, the rs1048661, which is the predominant SNP associated with XFS in East Asians, was not associated with the disease [31].

Therefore, this study was conducted to investigate the association between the three *LOXL1* SNPs and XFS in the Korean population. In addition, we investigated the association between the three SNPs and the phenotypic features of patients with XFS.

## Methods

### Study subjects

Eighty-nine unrelated patients diagnosed with XFS including XFG and 146 unrelated control subjects diagnosed with cataract, primary open angle glaucoma (POAG), chronic angle closure glaucoma (CACG), or secondary glaucoma were recruited from the Yeungnam University Hospital in Daegu, Korea. This study adhered to the tenets of the Declaration of Helsinki. The study protocol was approved by the Institutional Review Board of Yeungnam University Hospital, and informed consent was obtained from all study participants.

All subjects underwent comprehensive ophthalmic examinations including slit-lamp biomicroscopy, gonioscopy, and fundoscopy. Subjects with XFS were defined as those with exfoliation material on the anterior lens capsule and/or pupillary margin in mydriasis by slit lamp biomicroscopy. Secondary open-angle glaucoma due to XFS was defined, if elevated intraocular pressure (>20 mmHg), an open-angle angle, and characteristic glaucomatous optic neuropathy with compatible visual field loss were found with clinical evidence of XFS. Control subjects included those with cataract, POAG, CACG, and secondary glaucoma (e.g., uveitis-induced glaucoma, neovascular glaucoma) with no clinical signs of XFS. To avoid possible misclassification of latent XFS as controls, considering that XFS is a late-onset disorder, only individuals aged 60 years or older were recruited as controls.

### Analysis of *LOXL1* polymorphisms

Genomic DNA was extracted from whole blood and purified using the Qiagen QIAamp Blood Kit (Qiagen, Valencia, CA). The three *LOXL1* SNPs (rs1048661, rs3825942, and rs2165241) were amplified by polymerase chain reaction (PCR), directly sequenced, and genotyped. For PCR, two primer sets (for rs1048661 and rs3825942 in exon 1: 5′-GGT GTA CAG CTT GCT CAA CTC G-3′ and 5′-GTA GTA CAC GAA ACC CTG GTC GTA-3′; for rs2165241 in intron 1: 5′-CTG CTC TGG TCC TTA CCA GGT ACT-3′ and 5′-GTA GTG GCC AGA GGT CTG CTA A-3′) were used under PCR conditions. The PCR protocol was as follows: initial denaturation at 95 °C/15 min, followed by 35 cycles (94 °C/30 s, 62 °C 1 min 30 s, 72 °C/1 min 30 s), and a final extension at 72 °C/4 min. Amplified PCR products were electrophoresed in 2% agarose gel and stained with ethidium bromide and purified by the QIAquick PCR purification kit (Qiagen). Genotypes of the three *LOXL1* SNPs were determined by direct DNA sequencing, using the BigDye Terminator v3.1 Kit (Applied Biosystems, Foster City, CA) in an ABI PRISM^TM^ 3100 Genetic Analyzer (Applied Biosystems, Perkin-Elmer).

### Statistical analysis

Hardy–Weinberg equilibrium of the genotypic frequencies among cases and separately among controls was tested using the χ^2^ test in SAS Genetics (ver. 9.2; SAS Institute Inc., Cary, NC). Allele and genotype frequencies between cases and controls were compared by the χ^2^ test and odds ratio (OR) with 95% confidence interval (CI) to XFS was calculated by the logistic regression method. Allele and genotype frequencies between subgroups, i.e., XFS without glaucoma and XFG in cases, and cataract, POAG, CACG, and secondary glaucoma in controls were also compared using the methods mentioned above. Pairwise linkage disequilibrium (LD) analysis among the three *LOXL1* SNPs was performed, and individual haplotype and their estimated population frequencies were inferred using HAPLOVIEW (ver. 4.2; Daly Lab, Broad Institute, Cambridge, MA). Logistic regression analysis was used for evaluation of the effects of multiple covariates when comparing XFS cases and controls. The covariates were the risk genotypes of the three *LOXL1* SNPs, sex, diabetes, hypertension, retinal vein occlusion, cardiovascular disease, and cerebrovascular disease. As a diagnostic test for XFS, sensitivity (SE), specificity (SP), and positive and negative predictive values (PPV and NPV) of the risk alleles and genotypes for the three SNPs were calculated for assessment of their clinical values.

## Results

The mean age of 89 Korean patients, including 28 cases with XFS without glaucoma and 61 cases with XFG, was 71.7±7.7 years (age range 53 to 92) and for 146 Korean control subjects including cataract, POAG, CACG, and secondary glaucoma, the mean age was 72.9±6.4 years (age range 60 to 90). The gender distribution was 48 (53.9%) males and 41 (46.1%) females in cases, and 67 (45.9%) males and 79 (54.1%) females in controls. There were no significant differences in age and gender distribution between cases and controls (p=0.216, p=0.232, respectively). Demographic characteristics of the study subjects are shown in Table 1.

**Table 1:** Demographic characteristics of the study subjects.

** **	**Cases**		**Controls**		**p value**
Number of subjects	89	** **	146	** **	** **
Subgroups (number)	XFS	28	Cataract	90	** **
** **	XFG	61	POAG	32	** **
** **	** **	** **	CACG	19	** **
** **	** **	** **	Secondary glaucoma	5	** **
**Gender (%)**					
Male	48 (53.9%)	** **	67 (45.9%)	** **	0.232
Female	41 (46.1%)	** **	79 (54.1%)	** **	
**Age**					
Mean±SD	71.7±7.7	** **	72.9±6.4	** **	0.216
Range	53-92	** **	60-90	** **	

XFS, Exfoliation syndrome; XFG, Exfoliation glaucoma; POAG, Primary open angle glaucoma; CACG, Chronic angle closure glaucoma; SD, Standard deviation.

Distributions of allele and genotype frequencies for SNPs rs1048661, rs3825942, and rs2165241 are shown in Table 2. The genotype frequencies of the three *LOXL1* SNPs in control subjects were in Hardy–Weinberg equilibrium (HWE). The genotype frequencies of the SNP rs3825942 in cases were also in HWE. On the other hand, the genotype distributions of the SNPs rs1048661 and rs2165241 showed a slight deviation from the HWE. We confirmed the reproducibility of genotyping of rs1048661 and rs2165241 by repeating DNA sequencing. Previous studies have shown that deviation from HWE in affected individuals may be indicative of the presence of susceptibility loci.

**Table 2:** Allele and genotype association analysis between XFS/XFG versus all controls and XFS/XFG versus controls aged 70 years or above.

** **	**XFS/XFG (n=89)**	**All controls (n=146)**			**Controls aged 70 years or above (n=110)**		
**SNP**	**Count (proportion)**	**Count (proportion)**	**p value**	**OR (95% CI)**	**Count (proportion)**	**p value**	**OR (95% CI)**
rs1048661							
Allele							
T	165 (0.93)	188 (0.64)	5.744×10^−12^	7.02 (3.80–12.97)	135 (0.61)	5.423×10^−13^	7.99 (4.27–14.95)
G	13 (0.07)	104 (0.36)	** **	** **	85 (0.39)	** **	** **
Genotype							
TT	80 (0.90)	64 (0.44)	2.066×10^-12*^	11.39 (5.31–24.42)^*^	45 (0.41)	1.177×10^-12*^	12.84 (5.85–28.21)^*^
GT	5 (0.06)	60 (0.41)	** **	** **	45 (0.41)	** **	** **
GG	4 (0.04)	22 (0.15)	** **	** **	20 (0.18)	** **	** **
rs3825942							
Allele							
G	175 (0.98)	261 (0.89)	0.0003	6.93 (2.09–23.01)	193 (0.88)	6.950×10^−5^	8.16 (2.43–27.37)
A	3 (0.02)	31 (0.11)	** **	** **	27 (0.12)	** **	** **
Genotype							
GG	86 (0.97)	116 (0.80)	0.0003^*^	7.11 (2.10–24.08)^*^	85 (0.77)	9.441×10^-5*^	8.43 (2.45–28.98)^*^
GA	3 (0.03)	27 (0.18)	** **	** **	23 (0.21)	** **	** **
AA	0 (0.00)	3 (0.02)	** **	** **	2 (0.02)	** **	** **
rs2165241							
Allele							
C	175 (0.98)	265 (0.91)	0.0011	5.94 (1.35–11.71)	199 (0.90)	0.0011	6.16 (1.81–20.99)
T	3 (0.02)	27 (0.09)	** **	** **	21 (0.10)	** **	** **
Genotype							
CC	87 (0.98)	122 (0.84)	0.0008^*^	8.58 (1.97–37.16)^*^	92 (0.84)	9.910×10^-4*^	8.51 (1.92–37.76)^*^
CT	1 (0.01)	21 (0.14)	** **	** **	15 (0.14)	** **	** **
TT	1 (0.01)	3 (0.02)	** **	** **	3 (0.03)	** **	** **

SNP, single nucleotide polymorphism; XFS, Exfoliation syndrome; XFG, Exfoliation glaucoma; OR, odds ratio; The asterisks (*) indicate the p values and OR ratios derived from comparison of the specific genotype with all of the others, i.e., TT versus GT+GG at rs1048661, GG versus GA+AA at rs3825942, CC versus CT+TT at rs2165241.

While no significant differences in allele and genotype frequencies were found between the XFS subgroup and the XFG subgroup in cases, and among subgroups, including cataract, POAG, CACG, and secondary glaucoma in controls, significant differences in allele and genotype frequencies of the three *LOXL1* SNPs were observed between entire cases and control subjects. SNP rs1048661 was associated with XFS including XFG (p=5.744×10^−12^), with the at-risk T allele presenting in 93% of patients (OR=7.02, 95% CI=3.80–12.97), while the T allele was detected in 64% of control subjects. The genotype of rs1048661 was also associated with XFS including XFG (p=2.066×10^−12^), with genotype TT found in 90% of patients (OR=11.39, 95% CI=5.31–24.42), while the TT was detected in only 44% of control subjects. At rs3825942, the frequencies of the G allele and GG genotype at rs3825942 were significantly higher in cases than in controls (p=0.0003, OR=6.93, 95% CI=2.09–23.01; p=0.0003, OR=7.11, 95% CI=2.10–24.08, respectively). The allele C and the genotype CC of rs2165241 presented at significantly higher frequencies in cases than in controls (p=0.0011, OR=5.94, 95% CI=1.35–11.71; p=0.0008, OR=8.58, 95% CI=1.97–37.16, respectively).

Considering that XFS is a late-onset disorder, we compared the results of association analysis with controls including only patients aged 70 years or older to those of entire controls (Table 2). The T allele and TT genotype of rs1048661 and the G allele and GG genotype of rs3825942 presented at lower frequencies in controls including patients aged 70 years or older than entire controls, conferring an increased risk to XFS, while the C allele and CC genotype of rs2165241 showed similar frequencies between the two subgroups.

In addition, we compared the frequencies of *LOXL1* polymorphisms by dividing the phenotypes of XFS into either XFS without glaucoma or XFG and into either unilateral or bilateral involvement (Table 3 and Table 4). Patients with XFG did not show an increasing tendency of risk allele and genotype frequencies of the three SNPs, compared to those with XFS without glaucoma. And patients with bilateral involvement did not have an increasing tendency of risk allele and genotype frequencies of three SNPs, compared to those with unilateral involvement.

**Table 3:** Allele and genotype association analysis between controls versus XFS and controls versus XFG.

** **	**Control (n=146)**	**XFS (n=28)**			**XFG (n=61)**		
**SNP**	**Count (proportion)**	**Count (proportion)**	**p value**	**OR (95% CI)**	**Count (proportion)**	**p value**	**OR (95% CI)**
rs1048661							
Allele							
T	188 (0.64)	52 (0.93)	2.455×10^−5^	7.19 (2.53–20.44)	113 (0.93)	4.094×10^−9^	6.95 (3.38–14.27)
G	104 (0.36)	4 (0.07)	** **	** **	9 (0.07)	** **	** **
Genotype							
TT	64 (0.44)	24 (0.86)	4.906×10^-5*^	7.69 (2.54–23.28)^*^	56 (0.93)	1.840×10^-10*^	14.35 (5.43–37.91)^*^
GT	60 (0.41)	4 (0.14)	** **	** **	1 (0.02)	** **	** **
GG	22 (0.15)	0 (0.04)	** **	** **	4 (0.07)	** **	** **
rs3825942							
Allele							
G	261 (0.89)	55 (0.98)	0.036	6.53 (0.87–48.87)	120 (0.98)	0.002	7.13 (1.68–30.26)
A	31 (0.11)	1 (0.02)	** **	** **	2 (0.02)	** **	** **
Genotype							
GG	116 (0.80)	27 (0.96)	0.032^*^	6.98 (0.91–53.48)^*^	59 (0.97)	0.002^*^	7.63 (1.76–33.03)^*^
GA	27 (0.18)	1 (0.04)	** **	** **	2 (0.03)	** **	** **
AA	3 (0.02)	0 (0.00)	** **	** **	0 (0.00)	** **	** **
rs2165241							
Allele							
C	265 (0.91)	56 (1.00)	0.018	1.10 (1.06–1.14)	119 (0.98)	0.015	4.04 (1.20–13.58)
T	27 (0.09)	0 (0.00)	** **	** **	3 (0.02)	** **	** **
Genotype							
CC	122 (0.84)	28 (1.00)	0.021^*^	1.20 (1.11–1.29)^*^	59 (0.96)	0.009^*^	5.80 (1.33–25.38)^*^
CT	21 (0.14)	0 (0.00)	** **	** **	1 (0.02)	** **	** **
TT	3 (0.02)	0 (0.00)	** **	** **	1 (0.02)	** **	** **

SNP, single nucleotide polymorphism; XFS, Exfoliation syndrome; XFG, Exfoliation glaucoma; OR, odds ratio. The asterisks (*) indicate the p values and OR ratios derived from comparison of the specific genotype with all of the others, i.e., TT versus GT+GG at rs1048661, GG versus GA+AA at rs3825942, CC versus CT+TT at rs2165241.

**Table 4:** Allele and genotype association analysis between controls versus unilateral XFS/XFG and controls versus bilateral XFS/XFG.

** **	**Control (n=146)**	**Unilateral XFS/XFG (n=70)**			**Bilateral XFS/XFG (n=19)**		
**SNP**	**Count (proportion)**	**Count (proportion)**	**p value**	**OR (95% CI)**	**Count (proportion)**	**p value**	**OR (95% CI)**
rs1048661							
Allele							
T	188 (0.64)	131 (0.94)	1.044×10^−10^	8.05 (3.93–16.49)	34 (0.89)	0.001	5.04 (1.736–14.62)
G	104 (0.36)	9 (0.06)	** **	** **	4 (0.11)	** **	** **
Genotype							
TT	64 (0.44)	63 (0.90)	1.110×10^-10*^	11.53 (4.95–26.86)^*^	17 (0.89)	0.0001^*^	11.69 (2.59–52.62)^*^
GT	60 (0.41)	5 (0.07)	** **	** **	0 (0.00)	** **	** **
GG	22 (0.15)	2 (0.03)	** **	** **	2 (0.11)	** **	** **
rs3825942							
Allele							
G	261 (0.89)	137 (0.98)	0.002	5.52 (1.62–18.06)	38 (1.00)	0.026	NA
A	31 (0.11)	3 (0.02)	** **	** **	0 (0.00)	** **	** **
Genotype							
GG	116 (0.80)	67 (0.96)	0.002^*^	5.54 (1.62–18.86)^*^	19(1.00)	0.027^*^	NA^*^
GA	27 (0.18)	3 (0.04)	** **	** **	0 (0.00)	** **	** **
AA	3 (0.02)	0 (0.00)	** **	** **	0 (0.00)	** **	** **
rs2165241							
Allele							
C	265 (0.91)	139 (0.99)	0.0007	14.16 (1.90–105.32)	36 (0.95)	0.520	1.62 (0.37–7.20)
T	27 (0.09)	1 (0.01)	** **	** **	2 (0.05)	** **	** **
Genotype							
CC	122 (0.84)	69 (0.99)	0.0012^*^	13.57 (1.808–102.53)^*^	18 (0.95)	0.248^*^	3.19 (0.40–25.22)^*^
CT	21 (0.14)	1 (0.01)	** **	** **	0 (0.00)	** **	** **
TT	3 (0.02)	0 (0.00)	** **	** **	1 (0.05)	** **	** **

SNP, single nucleotide polymorphism; XFS, Exfoliation syndrome; XFG, Exfoliation glaucoma; OR, odds ratio; NA; non applicable. The asterisks (*) indicate the p values and OR ratios derived from comparison of the specific genotype with all of the others, i.e., TT versus GT+GG at rs1048661, GG versus GA+AA at rs3825942, CC versus CT+TT at rs2165241.

Pairwise linkage disequilibrium (LD) analysis showed that exonic SNPs, rs1048661 and rs3825942, were in complete LD (Coefficient of LD [D’]=1.00). SNP rs1048661 and intronic rs2165241 were also in strong LD (D’=0.87) while rs3825942 and rs2165241 were not in LD (D’=0.00). Haplotypes defined by consideration of LD of the three SNPs were estimated (Table 5). Seven of the eight theoretically possible haplotypes were detected in the haplotype analysis. The T-G-C haplotype was identified as having a significant association with XFS, conferring an approximately sevenfold increased risk to the disease (OR=6.61, 95% CI=3.58–12.22, p=3.352×10^−12^). The G-G-T haplotype, identified as a risk haplotype in Caucasians, was found to be protective in our study (OR=0.24, 95% CI=0.09–0.64, p=0.002). In addition, the haplotype G-G-C was observed only in cases, while the haplotype G-A-T was exclusively observed in controls.

**Table 5:** Haplotype association analysis between *LOXL1* SNPs and XFS/XFG.

**Haplotype**			**Proportion**				
rs1048661	rs3825942	rs2165241	**Case**	**Control**	p value*	OR* (95% CI)	Adjusted OR** (95% CI)
T	G	C	0.93	0.66	3.352×10^−12^	6.61 (3.58–12.22)	7.87 (4.10–15.11)
T	G	T	0.01	0.00	0.723	1.64 (0.10- 26.45)	1.46 (0.09- 23.67)
T	A	C	0.02	0.10	0.002	0.21 (0.07- 0.60)	0.20 (0.07–0.59)
T	A	T	0.01	0.07	0.002	0.08 (0.01- 0.61)	0.06 (0.01–0.49)
G	G	C	0.01	0.05	0.033	0.23 (0.05–1.01)	0.19 (0.04–0.86)
G	G	T	0.03	0.11	0.002	0.24 (0.09–0.64)	0.22 (0.08–0.60)
G	A	T	0	0.02	0.054	NA	NA

*LOXL1*, Lysyl oxidase-like 1; SNP, single nucleotide polymorphism; XFS, Exfoliation syndrome; XFG, Exfoliation glaucoma; OR, odds ratio; NA; non applicable. The single asterisk indicates the p values and OR ratios derived from comparison of the specific haplotype with all of the other haplotypes. The double asterisk indicates the OR values derived from comparison of each haplotype with other haplotypes, age, sex, RVO, DM, HTN, cardiovascular and cerebrovascular disease.

Step-wise logistic regression was used for simultaneous analysis of the effect of multiple covariates when comparing XFS/XFG patients with control subjects (Table 6). After adjusting for the effect of the risk genotypes of the three *LOXL1* SNPs and factors known to influence the prevalence of XFS, including sex, diabetes mellitus, hypertension, retinal vein occlusion, cardiovascular disease, and cerebrovascular disease, only rs1048661 among three SNPs remained significantly associated with disease risk (OR=12.13, 95% CI=4.11–35.78, p=6.113×10^−6^). Ischemic heart disease also showed a marginal association with disease risk (OR=7.75, 95% CI=1.41–42.51, p=0.018).

**Table 6:** Estimates of XFS/XFG risk from a logistic regression model (multivariate analysis).

**Regression term**	**OR**	**95% CI**	**p value**
rs1048661 TT versus TG+GG	12.13	4.11–35.78	6.113×10^−6^
rs3825942 GG versus GA+AA	1.13	0.23–5.50	0.878
rs2165241 CC versus CT+TT	2.93	0.52–16.38	0.222
Age/year	0.99	0.95–1.04	0.715
Sex (male versus female)	0.88	0.46–1.66	0.685
DM versus no DM	0.33	0.14–0.76	0.009
HTN versus no HTN	1.19	0.59–2.42	0.623
RVO versus no RVO	0.20	0.02–1.82	0.153
CVA+TIA versus no CVA+TIA	1.38	0.37–5.11	0.628
IHD versus no IHD	7.75	1.41–42.51	0.018

XFS, Exfoliation syndrome; XFG, Exfoliation glaucoma; OR, odds ratio; DM, Diabetes mellitus; HTN, Hypertension; RVO, Retinal vein occlusion; CVA, Cerebrovascular accident; TIA, Transient ischemic attack; IHD, Ischemic heart disease.

The risk alleles and genotypes of the three SNPs were analyzed for their ability to predict the affection status (Table 7). Although the SE was high, SP, PPV, and NPV were low for the risk alleles and genotypes of all three SNPs.

**Table 7:** Sensitivity, specificity, and positive and negative predictive values for the three *LOXL1* SNPs.

**SNP**	**SE (95% CI)**	**SP (95% CI)**	**PPV (95% CI)**	**NPV (95% CI)**
rs1048661				
Allele (T)	0.927 (0.889–0.965)	0.356 (0.285–0.427)	0.467 (0.393–0.541)	0.889 (0.843–0.935)
Genotype (TT)	0.889 (0.836–0.962)	0.562 (0.458–0.666)	0.556 (0.452–0.660)	0.901 (0.839–0.963)
rs3825942				
Allele (G)	0.983 (0.964–1.002)	0.106 (0.061–0.151)	0.401 (0.329–0.473)	0.912 (0.870–0.954)
Genotype (GG)	0.966 (0.928–1.004)	0.199 (0.116–0.282)	0.424 (0.321–0.527)	0.906 (0.845–0.967)
rs2165241				
Allele (C)	0.983 (0.964–1.000)	0.093 (0.050–0.136)	0.398 (0.326–0.470)	0.900 (0.000–0.944)
Genotype (CC)	0.978 (0.947–1.000)	0.164 (0.087–0.241)	0.416 (0.313–0.519)	0.923 (0.867–0.977)

SNP, single nucleotide polymorphism; SE, sensitivity; SP, specificity; PPV, positive predictive values; NPV, negative predictive values.

## Discussion

Since Thorleifsson et al. [18] reported results of a genome-wide association study of XFS, which identified three strongly associated polymorphisms of *LOXL1*, the association between XFS and the *LOXL1* gene has been confirmed in various Caucasian populations, including European, American, and Australian [21-25]. However, this association in the Asian population has been reported only in Indian, Chinese, and Japanese [26-31]. Thus, we investigated the association between *LOXL1* and XFS in the Korean population. In the current study, all three *LOXL1* SNPs, rs1048661, rs3825942, and rs2165241, were found to have a significant association with XFS.

Allelic and genotypic distributions of the three *LOXL1* SNPs were quite different between Caucasians and Asians. The risk for disease in Caucasians was associated mostly strongly with G allele and GG genotype of rs1048661, G allele and GG genotype of rs3825942, and T allele and TT genotype of rs2165241 [21-25]. However, Asians have shown different association results among different populations and districts. While the Indian study showed that the risk alleles and genotypes of rs1048661 and rs3825942 were consistent with those reported in Caucasians [30], studies from Japanese and Northern Chinese populations revealed that T allele and TT genotype of rs1048661 and C allele and CC genotype of rs2165241 were associated with XFS, providing reversed risk alleles and genotypes from Caucasians [26-29]. Moreover, in the Southern Chinese population, no association with XFS was found for rs1048661, which is the predominant SNP associated with XFS in Northern Chinese population [31]. Our study showed that T allele and TT genotype of rs1048661, G allele and GG genotype of rs3825942, and C allele and CC genotype of rs2165241 were significantly associated with Korean XFS patients. We found that the allelic and genotypic distributions of rs1048661 and rs2165241 in Korean have a similar pattern with those of East Asians, including Japanese and Northern Chinese, while they have a different pattern from those of Caucasians. However, the allelic and genotypic distribution of rs3825942 followed a similar pattern in all populations, including Caucasians and Asians. Exceptionally, a study in a black South African population reported that the risk at rs3825942 was the A allele not the G allele observed to increase risk in all other reported population [32]. The reasons for the discrepancy in the allelic and genotypic distributions of these *LOXL1* SNPs among XFS patients with different ethnicities remain unknown.

Considering that XFS is a late-onset disease, this study essentially included patients aged 60 years or older as control subjects. And, to reduce the chance of misclassifying latent or preclinical XFS into controls, only patients aged 70 years or older were re-grouped as control subjects. Comparison between XFS/XFG patients and control subjects aged 70 years or older revealed a more significant increase in the association of the *LOXL1* SNPs with XFS except for rs2165241 showing similar significance.

In the present study, the allelic and genotypic frequencies of the three *LOXL1* SNPs in the two XFS sub-phenotypes, i.e, XFS without glaucoma and XFG, were similar and showed no statistical significance. This finding is consistent with those of previous studies [18,27]. However, Schlötzer-Schrehardt et al. [20] reported decreased LOXL1 expression in cadaveric ciliary body specimens from XFG. Khan et al. [33] also demonstrated that *LOXL1* gene expression was reduced in lens capsule specimens from XFG but not XFS individuals and suggested a causative functional relationship between *LOXL1* expression and XFG. In addition, we compared the allelic and genotypic frequencies of the three *LOXL1* SNPs between the XFS sub-phenotypes divided into unilateral or bilateral involvement, which has not been evaluated so far. We found no significant difference in the comparison. Thus, it is difficult to explain these phenotype differences on the basis of the alleles and genotypes of *LOXL1*. These results suggest that *LOXL1* is more likely a contributing factor to disease onset of XFS, rather than a modifying factor for progression into several different phenotypes, and other genetic or environmental factors may be associated with phenotype differences in patients with XFS.

Using haplotype-based association analysis, the T-G-C haplotype, which was not associated with XFS in Caucasian populations, was the only risk haplotype in the Korean population, as it was in the East Asian populations including the Japanese and Northern Chinese [26,27,29]. Moreover, the G-G-T haplotype, which was the dominant haplotype in Caucasian populations [21,22,24], was a protective haplotype in our study. The frequency of the T-G-C haplotype as the major haplotype in the Korean population was 93%, which is consistent with 88%–94% reported in the East Asian population including the Japanese and Northern Chinese [26,27,29], whereas in Singaporean Chinese, the T-G haplotype defined by rs1048661 and rs3825942 was not associated with XFS [31]. This discrepancy observed in the risk haplotypes between East Asians and Singaporean Chinese may in part be caused by the effects of other unlinked modifier genes and/or environmental factors influencing disease penetrance in these two different populations.

After adjusting for the effect of risk genotypes of the three *LOXL1* SNPs and factors known to influence the prevalence of XFS, including sex, diabetes mellitus, hypertension, retinal vein occlusion, cardiovascular disease, and cerebrovascular disease, only rs1048661 among the three *LOXL1* SNPs showed an independent association with XFS in the Korean population, suggesting that the other two SNPs are not independent, but are more likely to be genetic markers in LD with rs1048661. Chen et al. [29] reported similar results that the association between the two SNPs, rs3825942 and rs2165241, and XFS was no longer significant after controlling for rs1048661 in the Northern Chinese population. Ozaki et al. [27] also showed that rs3825942 was not independent, but highly correlated with rs1048661 in the Japanese population. On the other hand, in an American study, rs1048661 was not associated with XFS after controlling for the other two SNPs [34]. So far, however, there has been no consolidated evidence to show which SNPs plays a prominent major role in the molecular pathogenesis of XFS. A recent study for investigation of genotype-correlated ocular expression of LOXL1 showed that LOXL1 expression was significantly increased in early XFS stages irrespective of the individual genotype [20]. These findings imply that other yet unidentified genetic and/or environmental factors unlinked to the *LOXL1* gene may be necessary to trigger the development of XFS.

Risk alleles and genotypes of the three *LOXL1* SNPs appeared to have high sensitivity; However, specificity and predictive values appeared low. Therefore, at present, genetic screening tests for these alleles and genotypes may be of limited usefulness.

In conclusion, we confirmed significant associations of the three *LOXL1* SNPs with XFS in the Korean population. While allelic, genotypic, and haplotypic frequencies differed between Korean XFS patients and Caucasian XFS patients, consistencies were observed in the allelic, genotypic, and haplotypic frequencies of the three SNPs among East Asian populations, including Korean, Japanese, and Northern Chinese. These *LOXL1* polymorphisms showed no significant correlation with the phenotypic features of XFS patients. The role of these SNPs in pathogenesis of XFS will require further study, and additional studies are also needed to unravel other genetic and/or environmental factors contributing to the different prevalence rates among ethnical populations and modulating the phenotypic expression of XFS.
